# The potential application of MR-derived ADCmin values from ^68^Ga-DOTATATE and ^18^F-FDG dual tracer PET/MR as replacements for FDG PET in assessment of grade and stage of pancreatic neuroendocrine tumors

**DOI:** 10.1186/s13550-023-00960-z

**Published:** 2023-02-08

**Authors:** Jing Gao, Si Xu, Huijun Ju, Yu Pan, Yifan Zhang

**Affiliations:** Department of Nuclear Medicine, Ruijin Hospital, Shanghai Jiao Tong University School of Medicine, No. 197, Ruijin 2nd Road, Shanghai, 200025 China

**Keywords:** ^68^Ga-DOTATATE, ^18^F-FDG, PET/MR, MR, Pancreatic neuroendocrine tumor

## Abstract

**Background:**

To evaluate the utility of ^68^Ga-DOTATATE and ^18^F-FDG PET/MR for prediction of grade and stage of pancreatic neuroendocrine tumors (PNETs), and to examine the correlation between parameters obtained from FDG PET and diffusion-weighted imaging (DWI) MR parameters.

**Methods:**

A retrospective study using ^68^Ga-DOTATATE and ^18^F-FDG PET/MR imaging was performed between April 2020 and May 2022 on 46 individuals with histologically confirmed PNETs. Metabolic tumor volume (MTV), maximum standardized uptake value (FSUVmax), and tumor lesion glycolysis (TLG) for FDG; somatostatin receptor density (SRD), maximum standardized uptake value (GSUVmax), and total lesion somatostatin receptor density (TLSRD) for DOTATATE; and minimum and mean apparent diffusion coefficient (ADCmin and ADCmean) values for MRI, respectively. We performed Spearman’s correlation analysis to examine the links between these variables and primary tumor stage and grading.

**Results:**

Higher PNET grading was associated with higher FSUVmax, MTV, and TLG values (*P* < 0.05). TLG, SRD, ADCmin, and ADCmean values were correlated with N staging, while SRD, MTV, ADCmin, TLG, and ADCmean were associated with M staging. Notably, ADCmin was a negative correlation between FSUVmax (*r* =  − 0.52; *P* < 0.001), MTV (*r* =  − 0.50; *P* < 0.001), and TLG (*r* =  − 0.56; *P* < 0.001).

**Conclusions:**

This study highlights significant correlative relationships between FDG PET-derived parameters and ADCmin. ADCmin may offer utility as a tool for PNET staging and grading in lieu of FDG PET. ^68^Ga-DOTATATE PET/MR alone may be a sufficient alternative to dual tracer PET/MR when conducting grading and staging of primary PNETs.

## Introduction

Pancreatic neuroendocrine tumors (PNETs) show great variation in their degrees of aggressiveness, their histological subtypes, and their clinical manifestations [1], resulting in the need for different treatment strategies and corresponding variability in patient prognostic outcomes [2].

The PNET categorization systems developed by the World Health Organization (WHO) and European Neuroendocrine Tumor Society (ENETS) are both widely used in clinical practice. The ENETS system entails the classification of PNETs and gastrointestinal NETs through the use of a four-stage TNM (tumor-node-metastasis) system, whereas the WHO system is based upon analyses of tumor cell proliferation through the detection of mitotic counts and Ki-67 expression levels. In recent reports, tumor grading and staging have been found to offer independent but complementary insight into the overall characteristics of a given malignancy [3, 4]. Noninvasive tumor grading and staging in particular would be of clinical benefit, which could reduce risks associated with invasive biopsy, aiding in preoperative assessments aimed at selecting the most optimal therapeutic strategy.

Currently, ^68^Ga-DOTA peptide-based imaging approaches are the gold standard used for assessing well-differentiated NETs exhibiting high levels of somatostatin receptor (SSTR) density [5]. However, since SSTR expression is lower on high-grade poorly differentiated NETs, ^18^F-FDG PET imaging is more reliable for evaluating these tumors [6]. ^68^Ga-DOTANOC PET-based SUVmax values can serve as a reflection of SSTR2 density [7], whereas ^18^F-FDG PET uptake values correspond to increases in metabolic activity at the cellular and tissue levels. Previous research efforts have demonstrated the complementary value of combining quantitative parameters derived from ^68^Ga-DOTA-peptides and ^18^F-FDG PET imaging analyses when evaluating PNET aggressiveness and conducting prognostic assessments [8, 9]. In contrast, apparent diffusion coefficient (ADC) values derived from diffusion-weighted imaging (DWI) from the MR sequence of PET/MR correlate with tumor cellularity such that they may offer value when evaluating tumor grading [10, 11]. While DWI and ^18^F-FDG PET offer insight into distinct molecular characteristics, glucose metabolic activity and tumor cellularity have also been reported to be positively correlated with one another [12]. When assessing tumor aggressiveness, the properties revealed by FDG and ADC are similar [13, 14]. However, evidence regarding the associations between MR and PET-derived parameters from PNET patient ^18^F-FDG and ^68^Ga-DOTATATE dual tracer PET/MR scanning is lacking at present. Therefore, this research was designed to further investigate the value of these various imaging measures in the preoperative grading and staging of primary PNETs.

## Materials and methods

### Patient selection

Our institution's ethics committee approved this retrospective research. Patients suspected of having PNET based on imaging results, clinical symptoms, and/or high tumor marker levels were recruited in this research between April 2020 and May 2022 and underwent ^68^Ga-DOTATATE PET/MR and ^18^F-FDG PET/MR scans retrospectively. Patients were included if they met the following criteria: a definitive biopsy- or surgical pathology-based histological diagnosis; adequate imaging quality for these analyses; and no previous therapy (surgery, radiotherapy, or chemotherapy). In total, 46 PNET patients were selected based on this criteria. The 2019 WHO classification system was used for tumor grading [15], while the ENETS system was used for tumor N and M staging [16].

### ^18^F-FDG and ^68^Ga-DOTATATE PET/MR analyses

Each patient had a PET/MR scan on two different days during a 2-week period. After fasting for at least 6 h, patients’ blood glucose levels were guaranteed to be below 11.1 mmol/L before the scan. ^18^F-FDG PET/MRI scans were performed 45–90 min after 2–5 MBq/kg ^18^F-FDG was injected intravenously (i.v.). After i.v. injection of 2.0 MBq/kg ^68^Ga-DOTATATE, PET/MRI scanning was commenced 45–60 min later. These studies used an integrated PET/MR scanner (Biograph mMR; Siemens Healthineers, Erlangen, Germany). Four different bed positions were used to acquire PET data from the middle of the thighs to the base of the skull (4 min each position), and then a single bed position was used to get data from the head (8 min total). While being scanned, patients remained supine with their arms at their sides. For the purpose of PET data reconstruction, a 3D attenuation-weighted ordered-subset expectation–maximization approach was used (2 iterations, 21 subsets, 256 × 256 matrices). The reconstructed images were smoothed using a Gaussian smoothing kernel that had a full width at half maximum (FWHM) of 6 mm. Cross-sectional T2-weighted 2D HASTE, echo-planar DWI (*b* values: 50 and 800 s/mm^2^), cross-sectional T1-weighted imaging, and T1WI-Dixon sequences were all collected concurrently with PET image collection. The ADC value was determined using a single exponential function (*b* values of 50 and 800 s/mm^2^).

### Quantitative image analyses

The LIFEx platform v5.1 [17] was used to extract all imaging parameters. Two nuclear medicine doctors, JG and SX, who had each worked with ^18^F-FDG and ^68^Ga-DOTATATAE PET/MR imaging for more than three years, hand-drew 3D volumes of interest (VOIs). Then, the ^18^F-FDG PET FSUVmax, MTV, and TLG parameters, as well as the ^68^Ga-DOTATATE PET maximum standardised uptake value (GSUVmax), somatostatin receptor density (SRD), and total lesion somatostatin receptor density (TLSRD) parameters, were taken from these primary tumors [8]. MTV was measured based on tumor segmentation using a 40% FSUVmax threshold, while TLG was defined as FSUVmean x MTV. SRD was measured based on tumor segmentation using a 40% GSUVmax threshold, with TLSRD similarly being defined as GSUVmean × SRD. The ADCmean and ADCmin values from primary tumors were measured using an MR ADC map while avoiding vessels, necrotic areas, and imaging artifacts.

### Statistical analysis

Continuous data that follow a normal distribution are represented as means and standard deviations (SD), whereas data that do not follow a normal distribution are reported as medians and interquartile ranges (IQR). Proportional frequencies are presented for categorical variables. Differences in tumor staging and grading were assessed with Mann–Whitney U tests, while binary variables were assessed with Fisher’s exact tests. Spearman rank correlation coefficients were used to analyze the correlation between imaging parameters and the Ki-67 index. Intraclass correlation coefficient (ICC) was used to analyze the inter-observer reproducibility. The imaging parameters associated with tumor grade and stage were analyzed using a forward logistic regression model (Wald method) in a multivariate analysis. This study used SPSS 26.0 (SPSS, IL, USA) with a significance threshold of *P* < 0.05 for all statistical analysis.

## Results

### Patient characteristics

Forty-six patients with histologically confirmed PNETs were scanned with ^68^Ga-DOTATATE PET/MR and ^18^F-FDG PET/MR between April 2020 and May 2022. The patients comprised 25 women (54.3%) and 21 men (45.7%), aged 22–75 y (median age, 52 y). Two of these 46 patients (4.3%) were diagnosed with multiple endocrine neoplasia type I syndrome. There were 29 patients with G1 tumors, 14 with G2 tumors, and 3 with NEC (a more severe kind of cancer) tumors. ENET N staging for these patients was as follows: N0 (*n* = 39), N1 (*n* = 7). ENET M staging for these patients was as follows: M0 (*n* = 36), M1 (*n* = 10). Table 1 provides a summary of the patient characteristics. The mean tumor size was 3.2 ± 2.8 cm (0.6–13.8 cm). A strong correlation was found between tumor size and MTV (*r* = 0.84, *P* < 0.001), TLG (*r* = 0.87, *P* < 0.001), SRD (*r* = 0.82, *P* < 0.001), TLSRD (*r* = 0.68, *P* < 0.001), and FSUVmax (*r* = 0.76, *P* < 0.001). Tumor size negatively correlated with ADCmin (*r* =  − 0.59, *P* < 0.001) and ADCmean (*r* =  − 0.44, *P* = 0.002). No significant correlation was found between tumor size and the GSUVmax (*r* =  − 0.04, *P* = 0.81).

**Table 1:** Clinical characteristics of PNET patients who underwent combined ^68^Ga-DOTATATE and ^18^F-FDG PET/MR

	Total, (%)
Gender, n (%)	
Female	25 (54.3)
Male	21 (45.7)
Mean age, years (range)	51 (22–75)
Primary site, n (%)	
Head/neck	14 (30.5)
Body/tail	29 (63.0)
Multifocal	3 (6.5)
WHO grade, n (%)	
G1	29 (63.0)
G2	14 (30.5)
G3-NEC	3 (6.5)
ENET stage, n (%)	
N stage	
N0	39 (83.0)
N1	7 (17.0)
M stage	
M0	36 (76.6)
M1	10 (23.4)

### Inter-observer reproducibility of ^68^Ga-DOTATATE, ^18^F-FDG PET, and ADC parameters

A high interobserver agreement level was demonstrated for ^68^Ga-DOTATATE PET parameters (GSUVmax:0.99, *P* < 0.001; SRD:0.82, *P* < 0.001; TLSRD:0.95, *P* < 0.001) and ^18^F-FDG PET parameters (FSUVmax:0.99, *P* < 0.001; MTV: 0.89, *P* < 0.001; TLG:0.95, *P* < 0.001). The ICC of ADCmin and ADC mean was 0.82 and 0.91 (all *P* < 0.001).

### Tumor grade, ^68^Ga-DOTATATE, ^18^F-FDG PET, and MR ADC relationships

Increases in the FSUVmax, MTV, and TLG ^18^F-FDG PET/MR parameters were observed with increasing tumor grade from G1 to G3, whereas GSUVmax values derived from ^68^Ga-DOTATATE PET/MR scans declined with tumor grade. SRD and TLSRD values rose with tumor grade. Significant decreases in ADCmin and ADCmean values were observed with increasing tumor grade. For further details regarding tumor characteristics, see Table 2.

**Table 2:** Parameters of ^68^Ga-DOTATATE and ^18^F-FDG dual tracer PET/MR based on tumor grade

Parameters	G1	G2	G3-NEC*	*P* (G1v G2)	*P* (G2 v NEC)	*P* (G1 v NEC)
FSUVmax	2.5 (2.1–7.3)	5.0 (2.8–9.3)	11.5 (8.1–13.6)	**0.04**	0.15	0.05
MTV	2.3 (1.1–6.1)	7.1 (2.5–28.4)	68 (51.6–804.3)	**0.04**	**0.03**	< **0.01**
TLG	3.8 (1.8–19)	24.8 (4.6–129.4)	505.7 (396.1–3168.3)	**0.03**	**0.03**	< **0.01**
GSUVmax	60.6 (37.8–99.5)	41.9 (19.0–72.4)	18.5 (4.5–23.4)	0.11	0.10	**0.02**
SRD	5.6 (3.1–20.6)	12.1 (6.7–44.8)	55.5 (27.5–705.9)	0.11	0.08	**0.02**
TLSRD	121.8 (60.3–427.2)	197.3 (52.8–1392.2)	236 (118.7–2597.1)	0.68	0.61	0.32
ADCmin	885.1 ± 140.4	703.4 ± 85.2	277.5 ± 79.2	< **0.01**	< **0.01**	< **0.01**
ADCmean	1485.2 ± 234.4	1322.0 ± 301.9	811.9 ± 79.4	**0.04**	< **0.01**	< **0.01**

Values are presented as median with interquartile range (IQR) in parentheses. The statistically significant differences are highlighted in bold

*SUV* Standardized uptake value, *MTV* Metabolic tumor volume, *TLG* Total lesion glycolysis, *SRD* Somatostatin receptor density, *TLSRD* Total lesion somatostatin receptor density

*Numbers in parentheses are ranges

### Ki-67 index, ^68^Ga-DOTATATE, ^18^F-FDG PET, and ADC parameters correlate

Ki-67 index values were significantly positively correlated with FSUVmax (*r* = 0.45, *P* < 0.01), TLG (*r* = 0.48, *P* < 0.01), MTV (*r* = 0.42, *P* < 0.01), and SRD (*r* = 0.43, *P* < 0.01), whereas they were significantly negatively correlated with ADCmin (*r* =  − 0.85, *P* < 0.01), GSUVmax (*r* =  − 0.33, *P* = 0.03), and ADCmean (*r* =  − 0.61, *P* < 0.01). Ki-67 index levels and TLSRD had a slight, nonsignificant connection (*r* = 0.19, *P* = 0.20).

### Relationships between tumor staging and ^68^Ga-DOTATATE, ^18^F-FDG PET, and ADC parameters

Increases in TLG (*P* = 0.03), SRD (*P* = 0.01), decreases in ADCmin (*P* < 0.01), and decreases in ADCmean (*P* < 0.01) were all associated with increasing tumor N staging. MTV (*P* = 0.03), TLG values (*P* = 0.03), SRD (*P* < 0.01), ADCmin (*P* < 0.01), and ADCmean (*P* < 0.01) were linked with tumor M staging. Tumor staging was not significantly linked with any of the other examined characteristics (*P* > 0.05) (Table 3).

**Table 3:** Parameters of ^68^Ga-DOTATATE and ^18^F-FDG dual tracer PET/MR based on N stage and M stage

Parameters	N0	N1	*P* (N0 v N1)	M0	M1	*P* (M0 v M1)
FSUVmax	2.7 (2.1–8.1)	5.9 (5.1–11.5)	0.07	2.6 (2.1–11.1)	5.3 (4.1–7.8)	0.17
MTV	2.6 (1.2–14.1)	25.3 (2.5–51.6)	0.07	2.5 (1.0–10.9)	22.1 (2.5–40.0)	**0.03**
TLG	4.5 (1.9–72.1)	96.8 (9.5–396.1)	**0.03**	4.3 (1.8–70.9)	60.3 (9.3–199.0)	**0.03**
GSUVmax	56.1 (24.5–85.1)	27.8 (16.1–95.1)	0.55	51.4 (25.3–89.5)	59.7 (14.3–83.0)	0.63
SRD	6.9 (3.1–20.5)	30.7 (17.3–70.4)	**0.01**	6.2 (3.0–22.7)	25.6 (11.1–91.9)	< **0.01**
TLSRD	121.8 (59.1–328.6)	264.4 (118.7–849)	0.24	115.3 (59.7–318.6)	271.7 (103.3–2689.6)	0.17
ADCmin	840.4 ± 152.7	510.4 ± 216.5	< **0.01**	843.0 ± 167.8	600.1 ± 201.8	< **0.01**
ADCmean	1446.8 ± 283.1	1084.1 ± 199.6	< **0.01**	1458.3 ± 282.0	1151.5 ± 246.2	< **0.01**

The statistically significant differences are highlighted in bold

### Relationships between ^18^F-FDG PET and MR parameters

**Fig. 1:**
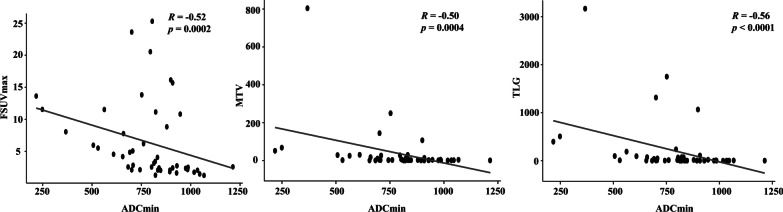
The correlation between ADCmin and FSUVmax, MTV, and TLG. *ADC* Apparent diffusion coefficient, *SUV* Standard uptake value, *MTV* Metabolic tumor volume, *TLG* Total lesion glycolysis

Correlation values of − 0.52 (*P* < 0.01), − 0.50 (*P* < 0.01), and − 0.56 (*P* < 0.01) were found between the ^18^F-FDG PET parameters FSUVmax, MTV, TLG and ADCmin, respectively (Fig. 1). There was no significant association between the parameters of ADCmean and ^18^F-FDG PET. These findings indicate that the ADCmin and FDG parameters have a negative connection, which may be predictive of PNET grade and staging.

### Preoperative risk factors for tumor grade and stage

Multivariate analysis revealed that only ADCmin could predict the presence of lymph nodal and distant metastases (*P* = 0.005 and *P* = 0.006, respectively). ADCmin could also differentiate between PNET G1 and G2/3 (*P* = 0.002). Other imaging variables were not significantly associated with tumor grade and staging.

## Discussion

The results of these analyses indicated that dual-tracer PET/MR can offer invaluable imaging data when conducting staging and grading assessments for PNET patients. Both tumor grade and Ki-67 index values were associated with several different parameters derived from dual-tracer PET/MR scans and were of value when predicting the M and N staging of primary PNETs. Strikingly, ADCmin was negatively correlated with FDG PET parameters (MTV, TLG, FSUVmax) in this study, suggesting that this PET/MR DWI-MR-derived ADCmin value can offer information equivalent to results derived from ^18^F-FDG PET scanning, thus potentially obviating the need for these ^18^F-FDG PET scans. As such, ^68^Ga-DOTATATE PET/MR alone rather than ^18^F- FDG and ^68^Ga-DOTATATE dual-tracer PET/MR is sufficient for PNET grading and stage.

Significant differences in both ^18^F-FDG uptake (MTV and TLG) and MR (ADCmin and ADCmean) parameters were observed when comparing PNETs of varying grades. Consistently, Ki-67 levels of primary neuroendocrine tumors were shown to have a positive connection between MTV and TLG levels by Abdulrezzak et al. [18]. Moreover, Chan et al. detected higher MTV values when assessing higher-grade tumors [19]. Increased Ki-67 expression is indicative of higher levels of cell proliferation, and the uptake of FDG is closely linked to this proliferative activity given that glycolytic activity is increased in rapidly proliferating cells such that they can generate the energy required for ongoing biosynthetic and replicative processes [20]. In other tumor types, correlations between Ki-67 index values and both ADCmin and ADCmean have been reported [21, 22], suggesting that these parameters are valuable biomarkers that reflect tumor biology in terms of proliferative activity. G3 PNETs in this study exhibited lower ^68^Ga-DOTATATE uptake levels as compared to G1 PNETs.

With respect to tumor staging, the present results revealed that the ^18^F-FDG-derived TLG and ^68^Ga-DOTATATE-derived SRD volumetric parameters from primary PNETs were capable of differentiating between different patient nodal (N0 and N1) and metastatic (M0 and M1) status. Moreover, MR-derived ADCmin and ADCmean values were significantly correlated with patient N and M staging. This could be a good help in identifying PNETs with malignant behavior. The potential utility of these PET/MR parameters has also been demonstrated in prior studies of PNET patients. For example, Langen et al. observed prognostic utility for both TLG and MTV in patients with high-grade gastroenteropancreatic neuroendocrine neoplasms [23]. De Robertis et al. observed a significant reduction in ADCmean values in individuals with stage III-IV lesions relative to individuals with lower tumor staging [24]. No associations were seen between SUVmax values obtained from ^18^F-FDG or ^68^Ga-DOTATATE PET/MR and N or M staging in patients with PNET. This may be attributable to the fact that SUVmax is based on a point-based tumor evaluation, whereas MTV can reflect the extent of the hypermetabolic tumor area. Higher numbers of hypermetabolic cells are more likely to correspond to nodal or distant metastasis. Moreover, in a multivariate analysis, the ADCmin of the primary tumor was the only significant predictor of tumor histological grading and stage, which could help predict tumor grade and stage for pretreatment tumor stratification.

Consistent with the present results, prior studies have not detected any significant correlations between ADCmean and FDG uptake parameters (FSUVmax, TLG, and MTV) [25, 26]. However, a correlation was detected between ADCmin values and MTV, TLG, and FSUVmax values, in line with prior studies on rectal cancer [27]. This is attributable to the fact that ADCmin values reflect the most highly proliferative areas of tumors and the regions with the greatest cell density [28]. As such, tumors exhibiting lower ADCmin values or higher FDG-derived TLG or MTV values are more likely to exhibit a higher Ki-67 index value and more advanced pathological staging. Moreover, the correlation between FDG parameters and ADCmin is relatively strong. Consequently, FDG and ADC values may have the potential to be interchangeably used when assessing the grading of PNET patients and evaluating tumor aggressiveness. Increased DOTATATE uptake indicates SSTR overexpression, which can identify patients to utilize peptide receptor radionuclide therapy (PPRT) [29] and may predict treatment response [30]. The DOTATATE and ADC values may provide complementary information to aid clinical decision-making and prognostication.

A key limitation of this study is the small size of the recruited patient population. As PNETs are a rare tumor type and dual-tracer PET/MR-based grading and staging of primary PNETs are only performed in some cases, however, recruiting a larger patient cohort will require future large-scale prospective research efforts. In addition, T staging was not taken into consideration in this study as they were high-grade PNET patients who could only undergo follow-up and were not eligible for surgical treatment.

## Conclusions

In summary, these analyses of ^18^F-FDG and ^68^Ga-DOTATATE dual-tracer PET/MR parameters in PNET patients revealed MR-derived ADCmin values to be negatively correlated with ^18^F-FDG-derived MTV, TLG, and FSUVmax values. As such, ^68^Ga-DOTATATE PET/MR scanning alone is likely to be sufficient as a means of assessing the grading and staging of primary PNET tumors.

## Data Availability

All data in our study are available from the corresponding authors upon reasonable request.

## Abbreviations

PNET: Pancreatic neuroendocrine tumor
PET/MR: Positron emission tomography/magnetic resonance
DWI: Diffusion-weighted imaging
ADC: Apparent diffusion coefficient
VOI: Volume of interest
SUVmax: Maximum standardized uptake value
MTV: Metabolic tumor volume
TLG: Total lesion glycolysis
